# Case Report: Visual Rehabilitation in Hemianopia Patients. Home-Based Visual Rehabilitation in Patients With Hemianopia Consecutive to Brain Tumor Treatment: Feasibility and Potential Effectiveness

**DOI:** 10.3389/fneur.2021.680211

**Published:** 2021-07-21

**Authors:** Monica Daibert-Nido, Yulia Pyatova, Kyle Cheung, Camilus Nayomi, Samuel N. Markowitz, Eric Bouffet, Michael Reber

**Affiliations:** ^1^Department of Ophthalmology and Vision Sciences, University of Toronto, Toronto, ON, Canada; ^2^Donald K. Johnson Eye Institute, Krembil Research Institute, University Health Network, Toronto, ON, Canada; ^3^Division of Hematology/Oncology, Research Institute, The Hospital for Sick Children, Toronto, ON, Canada; ^4^Laboratory Medicine and Pathobiology, Cell and System Biology, University of Toronto, Toronto, ON, Canada

**Keywords:** low-vision, rehabilitation, virtual-reality, personalized medicine 2, hemianopia or hemianopsia, hemianopia rehabilitation

## Abstract

**Background/Objectives:** Visual field loss is frequent in patients with brain tumors, worsening their daily life and exacerbating the burden of disease, and no supportive care strategies exist. In this case series, we sought to characterize the feasibility and potential effectiveness of a home-based visual rehabilitation program in hemianopia patients using immersive virtual-reality stimulation.

**Subjects/Methods:** Two patients, one with homonymous hemianopia and the other with bitemporal hemianopia, consecutive to pediatric brain tumors, with no prior visual rehabilitation performed 15 min of home-based audiovisual stimulation every 2 days for 6 weeks (case 2) and 7 weeks (case 1) between February and August 2020. Patients used a virtual-reality, stand-alone, and remotely controlled device loaded with a non-commercial audiovisual stimulation program managed in real time from the laboratory. Standard visual outcomes assessed in usual care in visual rehabilitation were measured at the clinic. Following a mixed method approach in this pragmatic study of two cases, we collected quantitative and qualitative data on feasibility and potential effectiveness and compared the results pre- and post-treatment.

**Results:** Implementation and wireless delivery of the audiovisual stimulation, remote data collection, and analysis for cases 1 and 2 who completed 19/20 and 20/20 audiovisual stimulation sessions at home, respectively, altogether indicated feasibility. Contrast sensitivity increased in both eyes for cases 1 and 2. Visual fields, measured by binocular Esterman and monocular Humphrey full-field analyses, improved in case 1. A minor increase was observed in case 2. Cases 1 and 2 enhanced reading speed. Case 2 strongly improved quality of life scores.

**Conclusion:** This is the first report of a home-based virtual-reality visual rehabilitation program for adult patients with hemianopia consecutive to a pediatric brain tumor. We show the feasibility in real-world conditions and potential effectiveness of such technology on visual perception and quality of life.

## Introduction

Central nervous system tumors are the second most common malignancies in childhood (1). Brain tumor and its treatment can affect the visual system at different levels, from the optic nerves (through compression or infiltration), to subcortical structures like the superior colliculus (SC) and lateral geniculate nuclei (LGN) to optic tracts, optic radiations, and visual cortices (1–4). Children with brain tumors can present visual impairments like decreased visual acuity and contrast sensitivity (CS), loss of color vision, and visual field loss such as hemianopias (1–5). These visual field defects in children affect their psycho-social and educational development with long-term debilitating effects. Hemianopia patients present difficulties in detecting stimuli in the defective visual field and show defective scanning and exploration (6). Moreover, they show a rotation and compression of the auditory space leading to imprecise localization of sound across both hemispaces (7). Patients with hemianopia naturally develop oculomotor strategies (more saccades toward the blind field) to compensate for visual field loss (8, 9), but visual rehabilitation procedure must still be developed to optimize/improve visual perception in the blind field and enhance the quality of life. Here, we report, for the first time, the feasibility and potential effectiveness of a visual rehabilitation procedure consecutive to a pediatric brain tumor in two young adult patients with hemianopia. We developed a home-based audiovisual stimulation protocol (currently not commercialized) using immersive virtual reality (IVR) in the stand-alone and remotely controlled head-mounted display (HMD) Oculus Go. The basic concept relies on the stimulation of residual vision and multisensory-induced visual plasticity using specific visual tasks, combined to spatially and temporally congruent auditory stimuli (10, 11). This approach is based on the multisensory integration properties of the brain for the detection and tracking of elements in the surroundings (12, 13). Recent evidence shows that static audiovisual stimulation reorganizes the functional connectivity in subcortical and cortical visual areas in hemianopia patients improving visual perception in the blind hemifield (11, 14). Here, we used the 3D-multiple object tracking (3D-MOT) paradigm, which closely matches attentionally demanding real-life situations (15, 16). The setup of the task is highly dynamic as the objects change their location over time, requiring a continuous deployment of visual attention and oculomotor control to avoid confusions between the objects (16). 3D-MOT stimulation programs displayed on monitors or TV screens have been shown to increase brain capacity for complex processes such as anticipation, eye-tracking, field of view, and even decision-making in healthy and pathological participants (17–19). Using a mixed method approach, our objective was to test the feasibility (treatment implementation and wireless delivery, remote collection and analysis of data, compliance and adherence, and adverse effects) and potential effectiveness (visual function and functional vision) of a home-based, IVR, audiovisual stimulation program on quality of life and visual field perception. This treatment was performed between March and August 2020, during the stay-at-home order (Ontario Government—Emergency Management and Civil Protection Act, Order in Council 518/2020) due to the COVID-19 pandemic. The audiovisual stimulation protocol was implemented remotely (telerehabilitation) in the patient's device and data were collected every 2 days. The patients were able to comply and adhere to the audiovisual rehabilitation protocol without any interruptions in the treatment.

## Case Description

### Case 1

**Figure 1:**
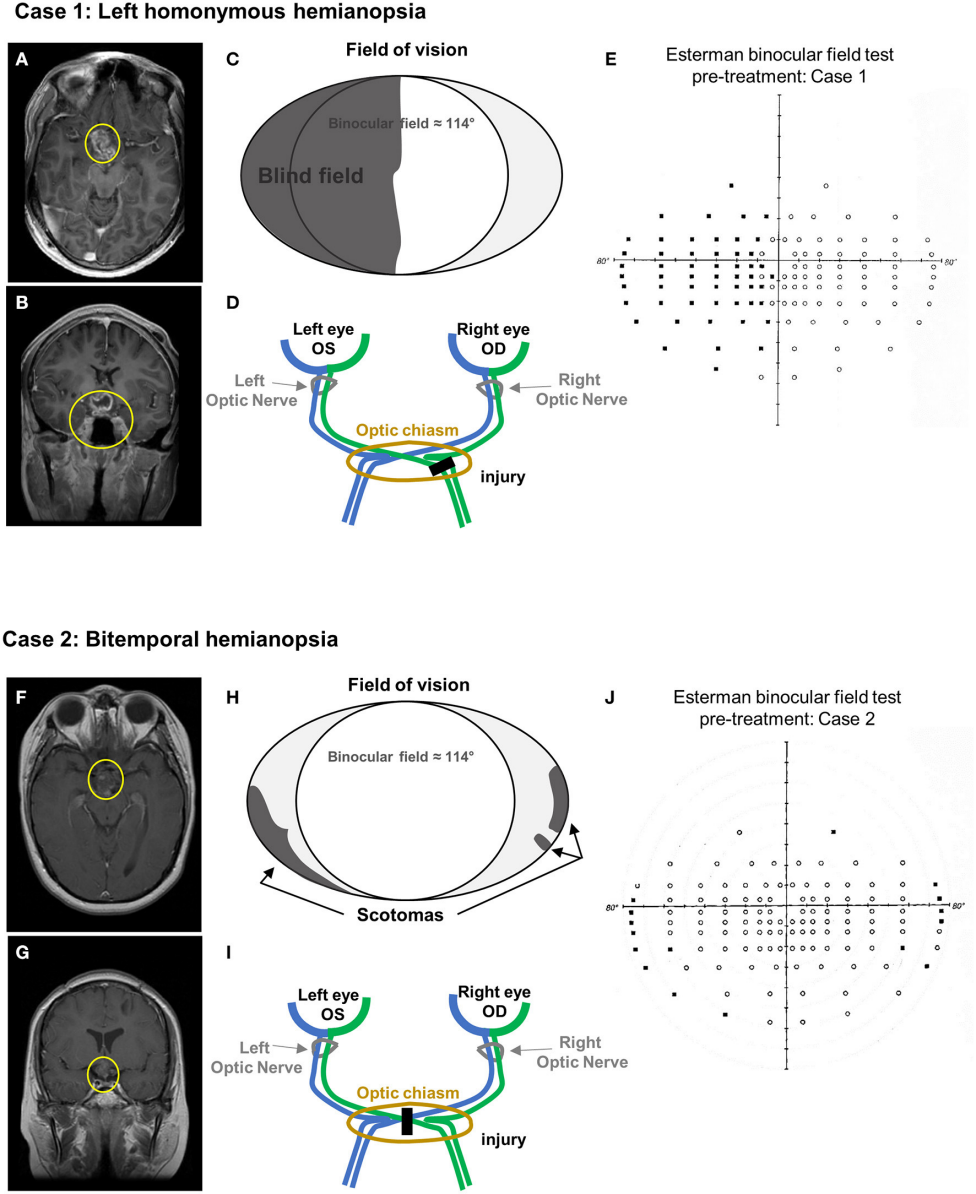
Case presentation. **(A–E)** Case 1 MRI axial **(A)** and coronal **(B)** view (T1 + contrast). Yellow circles indicate the remaining tumor mass after chemotherapy. **(C)** Schematic of the visual field defects. **(D)** Diagram of the optic pathway indicating the location of the injury. **(E)** Esterman binocular field test at baseline (pre-treatment). Black dots correspond to unseen point of light. **(F–J)** Case 2 MRI axial **(F)** and coronal **(G)** view (T1 + contrast). Yellow circles indicate the remaining tumor mass after chemo- and focal radiotherapy. **(H)** Schematic of the visual field defects. **(I)** Diagram of the optic pathway indicating the location of the injury. **(J)** Esterman binocular field test at baseline (pre-treatment). Black dots correspond to unseen point of light.

A 29-year-old male was diagnosed with a left homonymous hemianopia consecutive to a low-grade glioma diagnosed at the age of 8 (Figures 1A–E) (Table 1). Initial symptoms were related to hydrocephalus and MRI scan identified disseminated nodules in the brain, the cerebellum, and along the spinal cord. A biopsy of a spinal nodule was performed, and findings were in keeping with the diagnosis of low disseminated grade glioma. He was treated with chemotherapy with a good clinical and radiological response. At the age of 16, he progressively developed left homonymous hemianopia and his MRI scan showed tumor progression in the optic chiasm. There is a heterogeneous enhancing lesion involving the floor of the third ventricle hypothalamus and suprasellar cistern (Figures 1A,B). The pituitary stalk and optic chiasm cannot be differentiated from the mass, which are most likely infiltrated (Figures 1A,B). Despite further chemotherapy that prevented further tumor progression, his visual deficit remained unchanged, with mild impact on his daily activities (Figures 1C–E).

**Table 1:** Summary of the episode of care.

**Case 1**			
Timeline/age	8 years old (1999)	16 years old (2007)	29 years old (2020)
Episode of care	Grade glioma in brain, cerebellum, and spinal cord - Chemotherapy - Diagnosis of left homonymous hemianopsia	- Tumor progression in the optic chiasm - Left homonymous hemianopsia worsening	- No improvement of the left hemianopia - 7-week home-based visual rehabilitation program
**Case 2**			
Timeline/age	13 years old (2001)		32 years old (2020)
Episode of care	Diagnosis of suprasellar non-germinatous germ cell tumor - Chemotherapy and focal radiotherapy - Diagnosis of bitemporal hemianopia and left exotropia		- No improvement of bitemporal hemianopia - 6-week home-based visual rehabilitation program

### Case 2

A 32-year-old female was diagnosed with bitemporal hemianopia and left exotropia consecutive to a suprasellar non-germinatous germ cell tumor diagnosed at the age of 13 (Figures 1F–J) (Table 1). She was treated with a combination of chemotherapy and focal radiotherapy and achieved an excellent response. The end of treatment MRI still showed a 1.5-cm residual mass in the suprasellar region, inseparable from the hypothalamus and optic chiasm, which remained unchanged over time (Figures 1F,G). Her visual impairment, corresponding to a bitemporal hemianopia (Figures 1H–J), was detected at the time of initial diagnosis did not improve despite the successful management of her tumor.

### Assessments

Visual assessments investigating both visual function and functional vision were performed at the Ophthalmology Low vision Clinic at the Toronto Western Hospital (University Health Network, Toronto, Canada) following standard procedures in low-vision rehabilitation (20–23). These assessments include visual acuity [best corrected visual acuity (BCVA)] measured using the Early Treatment Diabetic Retinopathy Study (ETDRS) charts at 4 m and CS measured using the Functional Acuity Contrast Test (FACT). Retinal sensitivity (RS) and fixation stability [FS; bivariate contour ellipse area (BCEA), 63%] were measured using the Macular Integrity Assessment (MAIA) microperimeter (CentreVue, Padova, Italy). Field of vision was evaluated by the monocular Humphrey full field and Esterman binocular field of vision, which were assessed using the Humphrey Full Field Analyzer 3 standard automated perimeter (HFA 3, Zeiss, Heilberg, Germany). Quality of life was measured in two subdomains (orientation and mobility—reading) using the Veteran's affairs low-vision visual functioning questionnaire (LV-VFQ) (24). Reading speed was measured at critical print size using the Minnesota Low Vision Reading (MNREAD) test (25). Results were analyzed using the coefficient of repeatability (COR), specific to each assessment, to compare pre- and post-treatment data (26). The COR, also referred as the smallest real difference (SRD), quantifies an absolute reliability in the same unit as the assessment tool. The COR is directly related to the 95% limits of agreement proposed by Bland and Altman (27). It corresponds to the value below which the absolute differences between two measurements would lie within 95% of probability. Therefore, measurement values strictly different from COR indicate an effect of the treatment (26, 28).

## Diagnostic Assessment

### Treatment

**Figure 2:**
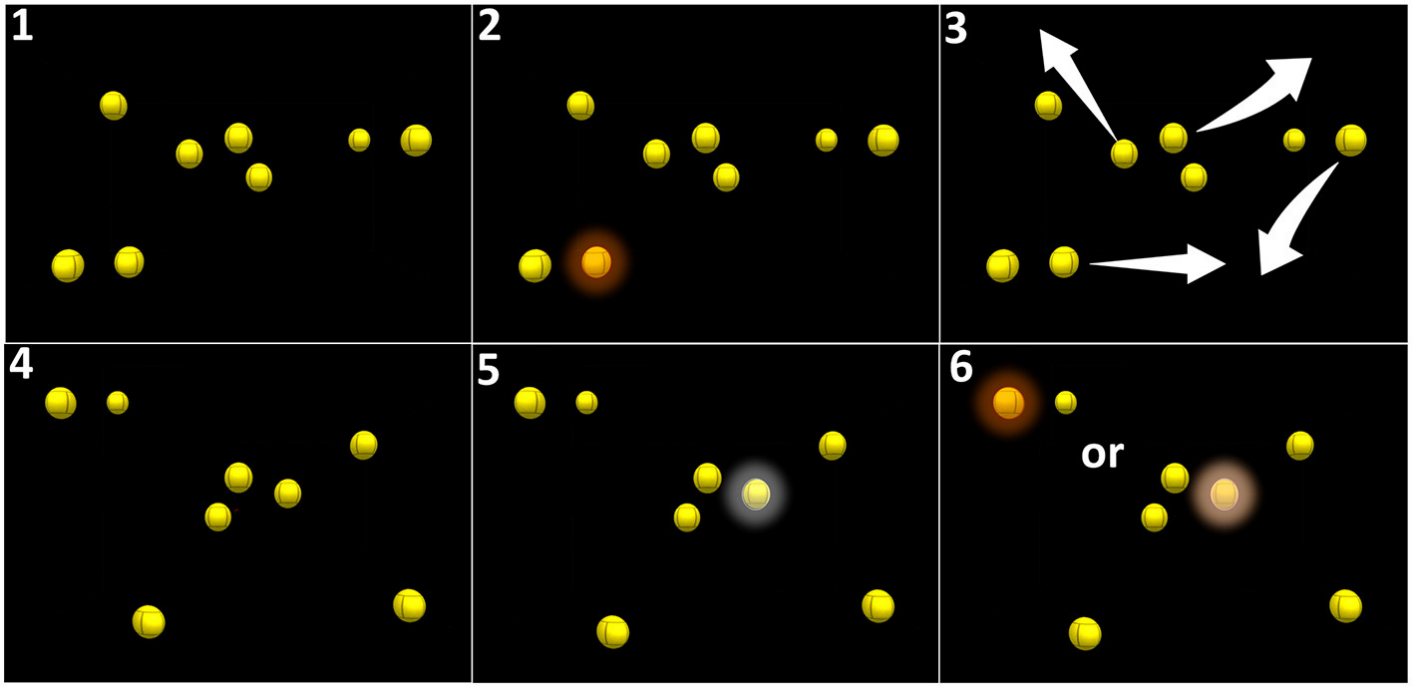
Principle of audiovisual stimulation program (NeurofyResearch). Sequence of the visual task. (1) Eight yellow still spheres are present in a virtual cube. (2) One of these spheres turns red for 5 s (cued target) and returns to yellow. (3) All spheres randomly move following linear paths across the visual field encompassing the blind field and bouncing on one another and on the walls of the virtual 3D cube when collisions occurred. (4) After 30 s, the spheres stopped moving. (5) The patient had to select the cued target using a hand-guided virtual laser pointer. (6) A correct selection is considered a positive hit.

The patients followed an IVR audiovisual stimulation protocol at home using the stand-alone and remotely controlled HMD Oculus Go for 7 weeks (case 1) or 6 weeks (case 2). Every 2 days, the patient performed one session of IVR audiovisual stimulation composed of three blocks of 15 trials of 20 s, equivalent to 3 × 5 min of continuous audiovisual stimulation. The audiovisual stimulation task corresponds to the 3D-MOT paradigm composed of eight high-contrast yellow spheres on a black background whose features were adapted to the visual ability of low-vision patients (luminosity = 100 cd/m^2^, size = 1.57° of visual angle). After one of the spheres was cued (turning red for 5 s and switching back to yellow, Figure 2), the spheres move for 20 s following random linear paths, bouncing on one another and on the walls of a virtual 3D cube when collisions occur. The overall span of the movement of the spheres covers 78° and 50° of horizontal and vertical visual angle, respectively (Figure 2). The initial speed of the spheres is adjustable, from 3°/s to 240°/s. and determined at baseline. A spatial sound (50 Hz, 25–65 dBHL) is correlated to the movement of the cued target. After 20 s, the movement stops and the patient is asked to select, using a virtual laser pointer, the cued sphere among the distractors (mark-all procedure) (16). If the selection is correct (i.e., corresponding to the cued target), a positive feedback sound is provided and the speed of the spheres in the next trial is increased by 0.05log. If the selection is incorrect (i.e., corresponding to a distractor), a negative feedback sound is provided and the speed of the spheres in the next trial is decreased by 0.05log (adaptative simple up-down staircase) (29). After each block of 5 min, the system sends data relative to the performance of the patient to our secured laboratory computer through Wi-Fi. Patient consent was obtained following UHN policies and according to the Declaration of Helsinki.

### Case 1

**Figure 3:**
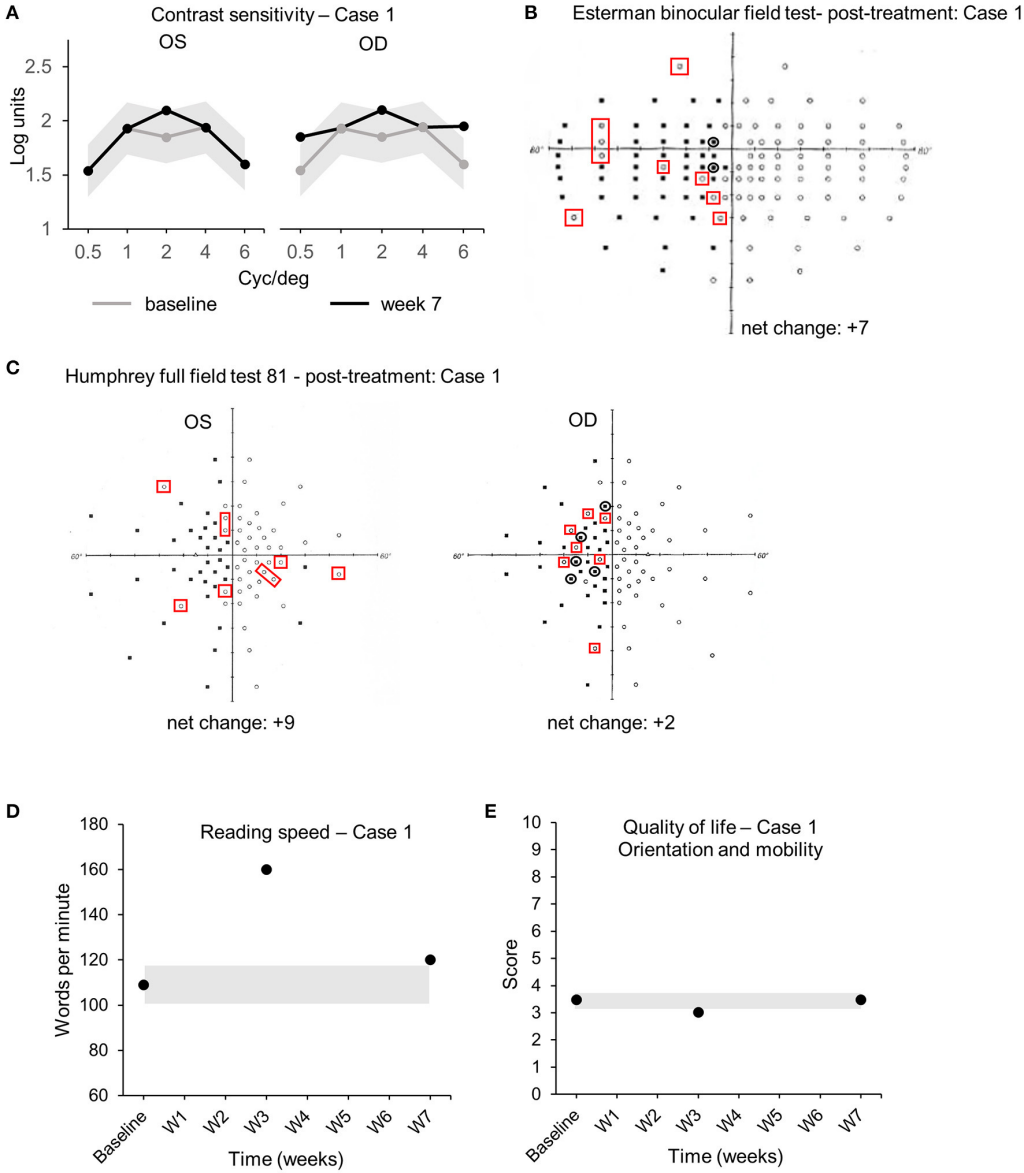
Case 1—Visual outcome measures. **(A)** Contrast sensitivity at baseline (gray line) and at the end of the treatment (week 7—black line) in the left (OS) and the right (OD) eye. **(B)** Esterman binocular field test post-treatment (week 7). Red squares indicate newly acquired points, black circles indicate lost points, compared to baseline. **(C)** Monocular Humphrey full-field test (81 points) post-treatment (week 7) in the left (OS) and right (OD) eye. Red squares indicate newly acquired points, and black circles indicate lost points, compared to baseline. **(D)** Reading speed at baseline, week 3, and post-treatment (week 7). **(E)** Quality of life scores at baseline, week 3, and post-treatment (week 7). Gray shaded areas in **(A,D,E)** indicate the values of the coefficient of repeatability (±COR). OD, *oculus dexter*; OS, *oculus sinister*; W, week.

The audiovisual stimulation parameters (initial speed, trial duration, number of targets, total number of spheres, number of blocks, and number of sessions) were updated and uploaded remotely into the patient's device from the laboratory's computers every 2 days for 7 weeks. Real-time data (date/time, average/maximum speed of the spheres, positive hits, number of trials/blocks/sessions performed, total time, and response time) were collected remotely from the device and indicated that case 1 completed the audiovisual stimulation protocol at home by performing 19 sessions of 3 blocks of 15 trials of 20 s each, every 2 days (±1 day), for a total continuous audiovisual stimulation of 4 h and 45 min. Visual assessments performed at the clinic comparing baseline (pre-treatment) and week 7 (post-treatment) demonstrated an enhanced CS in both left (*Oculus Sinister*, OS) and right (*Oculus Dexter*, OD) eyes. CS improved at 2 cyc/deg from 1.65 to 2.1 logits [COR ±0.24 logits (30)] in OS (Figure 3A). CS increased at 0.5, 2, and 6 cyc/deg for OD from 1.54 to 1.85 logits, from 1.85 to 2.1 logits, and from 1.6 to 1.95 logits [COR ±0.24 logits (30)], respectively (Figure 3A). Visual field analysis revealed that the number of points seen in the binocular Esterman field of vision test increased from 66 points seen at baseline to 69 at week 3 and 73 at week 7 with some reorganization (black circles indicate loss of points, red squares indicate acquired points, net change: +7 points, all in the blind left hemifield, +5.8% from baseline, Table 2, Figure 3B). Importantly, 3 adjacent points at 60° eccentricity in the left blind hemifield were detected at week 7, suggesting an improved peripheral visual perception in the blind field (Figure 3B red squares). Monocular visual field analysis using Humphrey full-field analysis (81 points) also revealed a valid (loss of fixation <20%) increase in the number of points seen in the full field with the left eye (OS) with an addition of 6 points between baseline (37/81) and week 3 (43/81) and +3 points between week 3 and week 7 (46/81) (net change: +9 points total, +5 in the blind left hemifield, +11.1% from baseline, loss of fixation 0/20–0%, Table 2, Figure 3C). There was a minor increase in the number of points seen in OD with some reorganization (net change: +2 points in the blind left hemifield, +2.5% from baseline, loss of fixation 1/19–5.3%, Table 2, Figure 3C). Reading speed improved from 109 words per minute (wpm) to 160 at week 3 and to 120 at week 7 [+10% between baseline and week 7, Figure 3D, black dots, COR ±8.6 wpm (31), Table 2]. No variations in visual acuity, RS, FS, or quality of life scores were observed between baseline, week 3, and week 7 (Table 2, Figure 3E). No adverse events were reported for the duration of the home-based stimulation.

**Table 2:** Case 1 baseline and after treatment outcome measures.

	**Baseline**		**Week 3**		**Week 7**		**COR**
	**OD**	**OS**	**OD**	**OS**	**OD**	**OS**	
Visual Acuity (VA)	20/20	20/25	20/20	20/25	20/20	20/25	
Retinal sensitivity (dB)	14.3	14.4	14	11.8	14.7	12.4	±1.51
Bivariate Contour Ellipse Area 63% (BCEA, 2)	0.4	0.6	0.5	0.5	0.3	0.5	±0.61
Humphrey Full Field	48/81	37/81	47/81 (−1)	43/81 (+6)	50/81 (+2)	46/81 (+9)	
(points seen)	(59.3%)	(45.7%)	(58.0%)	(53.1%)	(61.7%)	(56.8%)	
Esterman binocular field	66/120		69/120 (+3)		73/120 (+7)		±3 adjacent points
(points seen)	(55%)		(57.5%)		(60.8%)		
Reading speed (wpm)	109		160		120		±8.6
Quality of life (orientation and mobility)	3.48		3.01		3.48		±0.44

*OD, oculus dexter; OS, oculus sinister; dB, decibels; wpm, words per minute; COR, coefficient of repeatability*.

### Case 2

**Figure 4:**
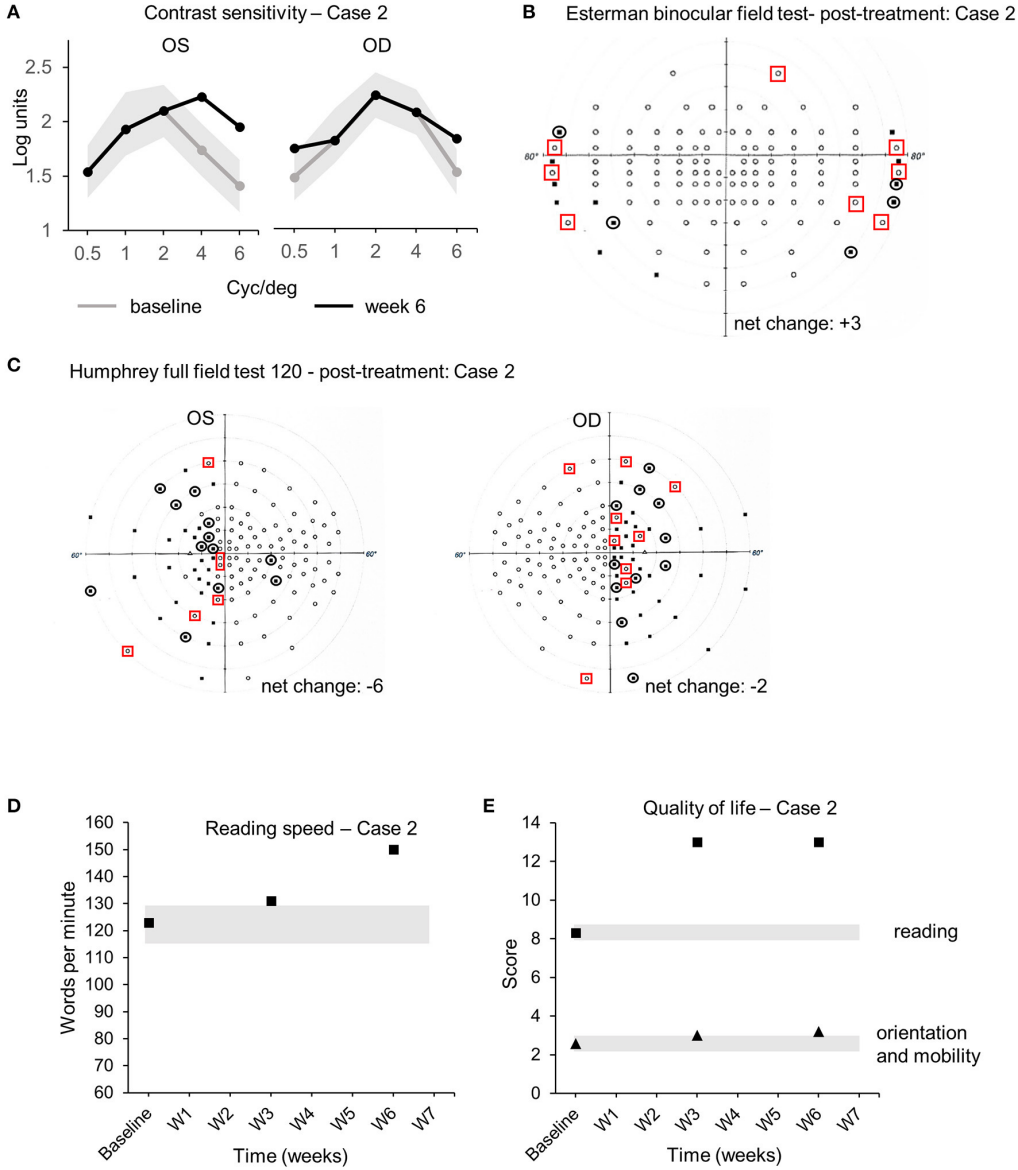
Case 2—Visual outcome measures and quality of life. **(A)** Contrast sensitivity at baseline (gray line) and at the end of the treatment (week 6—black line) in the left (OS) and the right (OD) eye. **(B)** Esterman binocular field test post-treatment (week 6). Red squares indicate newly acquired points, and black circles indicate lost points, compared to baseline. **(C)** Monocular Humphrey full-field test (120 points) post-treatment (week 6) in the left (OS) and right (OD) eye. Red squares indicate newly acquired points, and black circles indicate lost points, compared to baseline. **(D)** Reading speeds at baseline, week 3, and post-treatment (week 6). **(E)** Quality of life scores at baseline, week 3, and post-treatment (week 6). Gray shaded areas in **(A,D,E)** indicate the values of the coefficient of repeatability (±COR). OD, *oculus dexter*; OS, *oculus sinister*; W, week.

The audiovisual stimulation parameters (same as case 1) were updated and uploaded remotely into the home-based patient's device from the laboratory computers every 2 days for 6 weeks. Real-time data (same as case 1) were collected remotely from the device to the laboratory's computer and indicated that case 2 completed the audiovisual stimulation protocol at home by performing 20 sessions of three blocks of 15 trials of 20 s each, every 2 days (±1 day), for a total continuous audiovisual stimulation of 4 h and 50 min. Visual assessments performed at week 3 and week 6 were compared to baseline. CS improved at 4 and 6 cyc/deg from 1.74 to 2.23 logits and from 1.41 to 1.95 logits [COR ±0.24 logits (30)], respectively, in OS (Figure 4B). CS improved at 0.5 and 6 cyc/deg for OD from 1.54 to 1.85 logits and from 1.6 to 1.95 logits [COR ±0.24 logits (30)], respectively (Figure 4A). The Esterman binocular field testing showed a minor increase in the number of points seen between baseline and week 6 with some reorganization (black circles indicate loss of points, red squares indicate acquired points, net change: +3 points in the scotomas, +2.5% from baseline, Table 3, Figure 4B). Monocular visual field analysis using Humphrey full-field analysis (120 points) indicated a decrease in the number of perceived points for both OS (net change: −6 points, 4 of them in the blind left hemifield, −5% from baseline, loss of fixation 2/15–13.3%, Table 3, Figure 4C) and OD (net change: −2 points in the blind right hemifield, −1.6% from baseline, loss of fixation 2/17–11.7%, Table 3, Figure 4C). Such discrepancy (−6 net change in OS) is due to the unstable fixation of case 2 at baseline with >20% of fixation losses (4/17, 23%), decreasing the reliability of these particular measures (32). Clinically meaningful increase in reading speed was observed between baseline and week 6 [+27 wpm, +22%, COR ±8.6 wpm (31), Table 3, Figure 4D]. This improvement is corroborated by an increase in the quality of life score in the reading section of the questionnaire [+4.7 logits, COR ±0.44 logits (19), Table 3, Figure 4E—black squares]. The mobility section of the quality of life questionnaire showed only minor improvement [+0.62 logits, COR ±0.44 logits (22), Table 3, Figure 4E—black triangles]. No improvements in visual acuity, RS, and FS were observed between all time points (Table 3). No adverse events were reported for the duration the home-based stimulation program [VRISE questionnaire score = 33/35, >25 the threshold for cybersickness (33)].

**Table 3:** Case 2 baseline and after treatment outcome measures.

	**Baseline**		**Week 3**		**Week 6**		**COR**
	**OD**	**OS**	**OD**	**OS**	**OD**	**OS**	
Visual Acuity (VA)	20/20	20/40	20/20	20/40	20/20	20/40	
Retinal sensitivity (dB)	16.2	13.5	14.9	15.2	14	13.9	±1.51
Bivariate Contour Ellipse Area 63% (BCEA, 2)	0.5	2.4	0.2	2.4	0.4	2	±0.61
Humphrey Full Field (points seen)	83/120 (69.2%)	92/120 (76.7%)	80/120 (−3) (66.7%)	91/120 (−1) (75.8%)	81/120 (−2) (67.5%)	86/120 (−6) (71.7%)	
Esterman binocular field	104/120		103/120 (−1)		107/120 (+3)		±3 adjacent points
(points seen)	(86.7%)		(85.8%)		(89.2%)		
Reading speed (wpm)	123		131		150		±8.6
Quality of life (reading)	8.3		13		13		±0.44
Quality of life (orientation and mobility)	2.58		3.01		3.2		±0.44

*OD, oculus dexter; OS, oculus sinister; dB, decibels; wpm, words per minute; COR, coefficient of repeatability*.

## Discussion

This case series shows the feasibility of a home-based visual rehabilitation program using an audiovisual 3D-MOT stimulation paradigm in IVR HMD and potential effectiveness on visual perception in hemianopia patients. We were able to implement the program and update the stimulation procedures remotely from the laboratory's computer to the patients' device through Wi-Fi and to collect real-time data during the stimulation procedure performed at home. Patients were able to adhere and comply to the stimulation program performed at home without interruption of care and with no adverse events related to the use of IVR. A total of 4 h 45 min and 4 h and 50 min of audiovisual 3D-MOT IVR stimulation led to the improvement of several visual metrics. These results are unlikely due to a learning effect of the visual tests as baseline and after treatment assessments at the clinic were separated by a minimum of 3 weeks, above the learning effect time window shown to last for up to 1 week (34). Both cases showed improvement, although to various degrees, in the binocular field of vision, which is in line with the paradigm of the audiovisual 3D-MOT stimulation (15) where spheres moving at different speeds must be tracked into a virtual 3D cube encompassing 78° and 50° of horizontal and vertical visual angle, respectively. The net change (+3) observed in case 2 seems minor but the very few points originally not seen (16/120–19.3%) and their very peripheral location (Figure 1J) makes their restoration even more remarkable as field restoration becomes more challenging with increased eccentricity (35, 36). A significant improvement in monocular visual field (Humphrey full-field test) was observed in case 1 for the left eye, corresponding to the side of the blind hemifield, but not for the right eye. Whether this corresponds to an effect of the dominant eye is unknown, although it has been shown previously that eye dominance does not affect visual field test results (37, 38). The audiovisual stimulation program was performed binocularly, which may have favored better perceptual learning. The increase in the number of points seen during binocular test in cases 1 and 2 and monocular test in case 1 is unlikely due to compensatory eye movements or saccades. Fixation monitoring revealed the absence of loss of fixation above threshold (20%) during the automated Esterman in cases 1 and 2 and Humphrey full-field tests in case 1 validating the visual field measures. Monocular Humphrey field testing in case 2 revealed a decrease in the number of points seen after treatment, particularly for the left eye (net change −6). This discrepancy is likely due to unstable fixation during the visual field test at baseline for case 2. As mentioned above, visual field measures are reliable when the proportion of fixation loss (the inability to stabilize the gaze and to fixate the center of the visual field) is below 20% (32). At baseline, case 2 showed a high number of fixation loss (4/7–23%), above the reliability threshold of 20%; therefore, no conclusion can be made as to the potential effect of the audiovisual stimulation on monocular visual field in case 2. The high contrast of the visual stimulation task (yellow spheres with luminosity = 100 cd/m^2^ on black background) correlates with an increased CS observed for both eyes in each case. Accordingly, a significant increase in reading speed was observed in both cases, in line with improved reading speed associated to higher CS (39). A major improvement in reading speed was also reported by case 2 in the quality-of-life questionnaire, supporting the quantitative results obtained from the MNREAD test.

In this report, positive effects on visual perception and quality of life were observed after <5 h of audiovisual IVR stimulation, in sharp contrast with other studies indicating beneficial effects after a minimum of 40 h of static audiovisual training, although using a different setup and device (40). This suggests that dynamic audiovisual stimulation procedures might be more efficient on specific visual features compared to static audiovisual stimulation procedures previously described (40–42).

Some differences in visual field restoration were observed between case 1 and case 2. Although both patients show anatomical and structural defects at the optic chiasm, they present major differences in visual field loss with different areas affected. Case 1 presents a blind left hemifield with loss of central and peripheral fields whereas case 2 shows bitemporal hemianopia, which translates into scotomas at the periphery (>60°) of the visual field. In the binocular condition, case 1 shows more severe visual field defects than case 2 (compare Figures 3B, 4B). In our audiovisual stimulation protocol, the virtual space covered by the moving spheres has a visual angle of 78° horizontally and 50° vertically, which covers a large proportion of the blind left hemifield in case 1 but only a minor proportion of the scotomas in case 2, which may lead to less peripheral stimulation in this particular case. The overall improvement in case 1 is stronger than in case 2 because the original binocular field defects were also more pronounced in case 1.

Visual field restoration in hemianopia patients has been a controversial topic (10, 43–46). Several studies suggest that visual fields can be restored in hemianopia by enhanced oculomotor function and compensatory eye movement (40, 41, 47). Others suggest that a restoration of visual perception in the blind hemifield could be the consequence of a functional reorganization of the connectivity in subcortical and cortical structures after visual rehabilitation (11, 14). Moreover, recent evidence indicates that audiovisual stimulation, during which auditory and visual stimuli are spatially and temporally correlated, improve visual perception more efficiently than visual-only stimulations (11, 45, 48). The exact mechanisms of visual field restoration are still largely unknown. Visual field restoration following a dynamic audiovisual stimulation may involve both neuronal plasticity in subcortical and cortical visual brain areas (11, 14, 49, 50) and oculomotor enhancement and compensatory eye movement (8, 47) in a non-mutually exclusive manner. In our protocol, the patients track a high-contrast, sound-generating, cued target using vision and audition. This tracking requires eye movement control and audiovisual processing, which may help to recalibrate audiovisual perception through neuroplasticity and, concomitantly, to stimulate oculomotor control. When traveling through the blind field or scotomas, the cued target can still be tracked using correlated spatial sound, reinforcing the audiovisual association. Considering that hemianopia patients often present impaired sound localization (7), such audiovisual stimulation program may also improve spatial sound localization. Moreover, repeated exposure to identical stimuli in an identical environment maximizes perceptual learning (51).

Our study shows limitations as to the extent of the visual field stimulated (78° horizontal and 50° vertical), which should be increased to approach the normal extent of the visual field (200° horizontal and 130° vertical) to further stimulate peripheral vision. An increase of the duration of the stimulation program (>7 weeks) with more sessions per day (2 sessions of 15 min separated by a few hours of rest to avoid VR induced symptoms) should be developed. Positive effects of the treatment were observed mostly in binocular vision (Esterman binocular field test) with less effect on monocular vision (Humphrey full-field test). This can be explained by the improved FS, which occurs under binocular condition (35) allowing more points to be seen with the Esterman binocular field test. Our audiovisual stimulation protocol is binocular, which very likely impacts binocular vision. A monocular version of our audiovisual stimulation procedure, tailored to the ability of each eye individually, should be tested. When visual field measurements are deemed not reliable, they should be repeated within a short time window (<24 h) to avoid unreliable data and to allow the patient to rest. The use of a built-in eye-tracking system in the device could provide valuable information as to the visual tracking strategy used by patients and would allow the elaboration of more powerful audiovisual stimulation programs.

IVR in HMD is an emerging and very promising visual rehabilitation approach using high-technology devices (52–56). It is developed to provide perceptual learning with better ecological validity due to virtual reality, greater flexibility due to home-based stimulation protocol, and improved efficiency due to patient-tailored protocols (52, 53). Our dynamic IVR audiovisual stimulation procedure based on the 3D-MOT paradigm mimics real-life, complex dynamical situations where individuals apprehend their environment using both visual and auditory information (16).

Although no conclusion related to effectiveness of our treatment can be made from this report, our results support the feasibility of a larger RCT and suggest that patients with long-term visual field defects are still be able to restore some visual perception, in line with more recent work indicating that adaptation and plasticity can still occur at later stages in adults (57). This case series started just before the first stay-at-home order in Ontario and continued throughout the COVID-19 pandemic without interruption of care of patient-tailored treatment, further supporting the relevancy of home-based, remotely controlled, rehabilitation procedures. Future pragmatic randomized controlled studies will address the effectiveness of audiovisual IVR rehabilitation procedure in adults and earlier during the disease in children when neuronal plasticity is more effective. Integrated knowledge translation and a patient-centered approach will be included in further RCTs using patients' and caregivers/partners' open-ended questionnaires. An estimation of long-term effectiveness of IVR audiovisual stimulation must also be addressed. A home-based visual rehabilitation procedure is a promising approach not only to decrease the burden of disease by substantially diminishing the number of visits to the clinic and therefore relieving the constraints of commute and transportation, but also for populations living remotely and often left without easy access to modern medicine and treatments.

## Patient Perspective

### Case 1

At the age of eight, I was diagnosed with brain and spine tumors. The primary site was on the optic chiasm, where the optic nerves connect to the brain. I underwent 2 years of chemotherapy, followed by another year when I was 19. The location of the tumors caused my vision to be impacted. For as long as I can remember, I have lived with a deficit in my left peripheral vision. This is something I was always told could never be fixed, that I would have to live with it for my entire life, and just have to learn to compensate for the deficit. So, I did, looking to my left more often than my right, though this did not always prevent me from bumping into objects or people while walking. I thought this was my new normal, and I would live the rest of my life with the left-side deficit. You can imagine my surprise when one of my doctors told me about the work being done by Dr. Reber. I had doubts anything could work but gave it a try anyway. I was told to use an oculus VR headset every other day, with a specialized program to assist my peripheral vision. To my surprise and excitement, after 3 months of doing this regimen, I could feel the positive effects from the VR headset. I am now much more confident walking around outside, bumping into things less, and can certainly notice the improvement in my left peripheral vision. I am extremely thankful for everything Dr. Reber has done to improve my quality of life, especially after being told this is something that could not be fixed. I am also grateful to donors such as yourselves who help to make this research possible. My life has been changed for the better as a direct result of Dr. Reber's work and will forever be grateful for that.

## Data Availability Statement

The original contributions presented in the study are included in the article/supplementary material, further inquiries can be directed to the corresponding author/s.

## Ethics Statement

Ethical review and approval was not required for the study on human participants in accordance with the local legislation and institutional requirements. The patients/participants provided their written informed consent to participate in this study. Written informed consent was obtained from the individual(s) for the publication of any potentially identifiable images or data included in this article.

## Author Contributions

MD-N, SM, EB, and MR designed the protocol, analyzed the data, interpreted the results, and wrote/edited the manuscript. MD-N, YP, and SM performed the assessments and generated the data. KC managed the remote control of the device. KC, CN, and MR extracted the data. All authors attest that they meet the current ICMJE criteria for Authorship.

## Conflict of Interest

The authors declare that the research was conducted in the absence of any commercial or financial relationships that could be construed as a potential conflict of interest.
